# Organic Amendments Modulate Soil Microbiota and Reduce Virus Disease Incidence in the TSWV-Tomato Pathosystem

**DOI:** 10.3390/pathogens9050379

**Published:** 2020-05-14

**Authors:** Giuliano Bonanomi, Daniela Alioto, Maria Minutolo, Roberta Marra, Gaspare Cesarano, Francesco Vinale

**Affiliations:** 1 Department of Agricultural Sciences, University of Naples Federico II, Portici, 80055 Naples, Italy; giuliano.bonanomi@unina.it (G.B.); alioto@unina.it (D.A.); maria.minutolo@unina.it (M.M.); gaspare.cesarano@unina.it (G.C.); 2 Task Force on Microbiome Studies, University of Naples Federico II, 80055 Naples, Italy; 3 Department of Veterinary Medicine and Animal Production, University of Naples Federico II, 80137 Naples, Italy; frvinale@unina.it; 4 Institute for Sustainable Plant Protection, National Research Council, Portici, 80055 Naples, Italy

**Keywords:** conventional farming, soil microbiome, enzyme-linked immunosorbent assay (ELISA) test, disease suppression, biological control

## Abstract

Application of organic amendments is considered an eco-friendly practice to promote soil fertility and suppressiveness against a wide range of soil-borne pathogens. However, limited information is available about the capabilities of organic amendments to control virus disease. In this study, the suppressiveness of different organic amendments (i.e., compost manure, biochar, alfalfa straw, and glucose) was determined against the *Tomato spotted wilt virus* (TSWV) on tomato plants in a 1-year-long mesocosm experiment. Organic treatments were compared to the ordinary soil management based on mineral fertilizers and fumigation. Tomato seedlings were inoculated with TSWV and the infection and symptoms were assessed three weeks later. The disease incidence was higher in soil treated with mineral fertilizers and fumigation (>80%) compared to the application of organic amendments, with alfalfa straw and biochar recording the lowest incidence (<40%). Moreover, soil microbiota structure and diversity were assessed by high-throughput sequencing of bacterial and eukaryotic rRNA gene markers. Several members belonging to the bacterial phyla of Acidobacteria, Actinobacteria, Bacteroidetes, and Proteobacteria, as well as members of the fungal genus *Acremonium*, were positively associated with plant health. This study showed that conventional practices, by shifting microbiome composition, may increase TSWV incidence and severity.

## 1. Introduction

Application of organic amendments (OAs), like compost, agro-industry wastes, green and animal manure, and biochar, is considered a practice of pivotal importance to sustain soil fertility, functions, and, consequently, crop production [1,2,3]. Beneficial effects resulting from OA applications include the improvement of soil structure [4], aggregation [5], water holding capacity [6], nutrient storage, and cycling [7]. In addition, applications of OAs affect the biological properties of soil like the development of soil microbial biomass and metabolic activity [8], as well as the richness and diversity of the microbial community [9,10]. Several studies reported that OAs, by modifying soil microbiota, can increase soil suppressiveness against numerous plant pathogens, including oomycetes (e.g., *Pythium* spp., *Phytophthora* spp.), fungi (e.g., *Botrytis cinerea*, *Fusarium oxysporum*, *Verticillium dahliae*, *Rhizoctonia solani*), and bacteria (e.g., *Ralstonia solanacearum*, *Pseudomonas syringae* pv. tomato) [11,12,13,14,15]. In contrast, little information is available about the capabilities of OAs to control virus diseases.

OAs application can control soil-borne pathogens directly, through the release of fungitoxic compounds [16,17,18], or indirectly by promoting the development of a suppressive microbiome [19,20]. It has been well documented that several soil microorganisms, which are referred to as plant growth-promoting rhizobacteria (PGPR) and fungi (PGPF), not only promote plant growth, but also play a crucial role in protection against pathogens [21,22,23]. Competition for nutrients and space [20], direct parasitism [24], and antagonism through the production of secondary metabolites (e.g., antibiotics) [25] are the main mechanisms used by beneficial microbes to control soil-borne pathogens. However, they can also act indirectly by inducing a systemic resistance response (ISR) in the host plants [26]. *Tomato spotted wilt virus* (TSWV) is a quasi-spherical tripartite, negative/ambisense, single-stranded RNA virus belonging to *Orthotospovirus* genus. The worldwide distribution and the capability to infect more than 1300 plant species make TSWV one of the most economically important plant viruses [27,28]. In the Mediterranean basin, TSWV, which is transmitted by thrips, is responsible for severe crop losses, especially in tomato and pepper productions [29,30]. Unfortunately, chemical control measures cannot be extensively used on plant viruses. For insect-transmitted viruses, the control of potential vectors and the use of genetically resistant cultivars represent the most common strategies to limit virus diseases. Moreover, repeated applications of insecticides to control vectors leads to the emergence of resistance or tolerance to chemicals. At the same time, mutations in the virus genome are frequent and may result in the breakdown of plant resistance [31]. In the last years, substantial advancements in biological control of thrips have been achieved with the use of predatory mites, insects, and beneficial microbes [32,33]. However, the development of alternative strategies to control virus diseases transmitted by insects, such as TMWV, is highly recommended.

The aim of this study was to explore the ability of OAs to control phytopathogenic viruses, using a TSWV-tomato pathosystem. In a previous work, we used a metagenomic approach to evaluate the effects of conventional (i.e., use of synthetic fertilizers and fumigants) versus organic (i.e., use of different organic amendments) soil management practices on soil microbiota [34]. Combined applications of green manure and biochar substantially changed the composition of the soil microbiome compared to the untreated control, or to the use of mineral fertilizers and soil fumigants. Here, tomato plants were cultivated in soil treated with different OAs or mineral fertilizer and synthetic fumigants and, subsequently, were artificially inoculated with TSWV. The development of disease symptoms was monitored for three weeks in order to evaluate the efficacy of different OAs to control TSWV. Moreover, the relationship between soil microbiota composition and TSWV incidence and severity was investigated by using a high-throughput sequencing of bacterial and eukaryotic rRNA gene markers.

## 2. Results

### 2.1. Effect of OAs on TSWV Inoculated Plants

**Figure 1:**
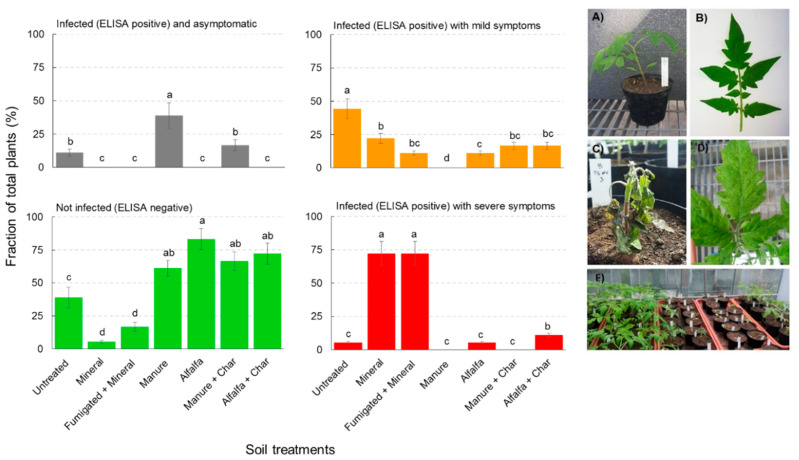
Effect of organic amendments on *Tomato spotted wilt virus* (TSWV)-inoculated plants. Left panel: tomato plant status at 21 days post inoculation with TSWV. Data are calculated from n = 18 replicates per treatment. Different letters indicate significant differences within each category according to one-way analysis of variance (ANOVA) followed by Duncan’s test at the 0.05 alpha-level of confidence. Statistical details are reported in Supplementary Tables S1 and S2. Right panel: selected pictures of tomato plants at 21 days post inoculation: (**A**,**B**) plants inoculated with TSWV, but not infected (enzyme-linked immunosorbent assay—ELISA-negative), (**C**) collapsing plant, (**D**) plant showing mosaic symptoms, (**E**) from left to right, plants not infected, collapsing, and stunted plants.

The occurrence of TSWV and the development of symptoms on tomato plants were evaluated at three weeks post inoculation, resulting in the plant status reported in Figure 1 and Supplementary Tables S1 and S2. The fraction of not infected (enzyme-linked immunosorbent assay—ELISA-negative) plants was significantly higher compared to the untreated control in all the organic-amended soil treatments, with the highest values (83.3% of total plants) observed in the case of Alfalfa treatment (Figure 1). Moreover, the fraction of not infected plants was lower in the case of Mineral or Fumigated + Mineral treatments (about 5.6% and 16.7% of total plants, respectively) compared to the untreated control (38.9% of total plants). The fraction of infected plants (ELISA-positive) with severe symptoms was very high in the case of Mineral and Fumigated + Mineral treatments (>70% of total plants), while all organic treatments recorded values not different from the control, except for Alfalfa + Char treatment (Figure 1). The fraction of infected plants (ELISA-positive) with mild symptoms reached 44.4% of total plants in the untreated control, while lower values were observed for the other treatments (ranging between 11% and 22% of total plants, up to 0% in the case of Manure treatment). Finally, the fraction of infected (ELISA-positive) but asymptomatic plants was the highest in Manure treatment (38.9% of total plants), but no significant differences between the untreated control and Manure + Char treatment were observed, nor were asymptomatic plants found in the other cases (Figure 1). Viral titer, assessed by ELISA as mean optical density (OD) value at 405 nm, was significantly lower in Alfalfa-amended plants, while no differences among other treatments were observed (Supplementary Tables S3 and S4).

### 2.2. Relationship Between Soil Microbiota Composition, TSWV Incidence, and Symptom Expression

**Figure 2:**
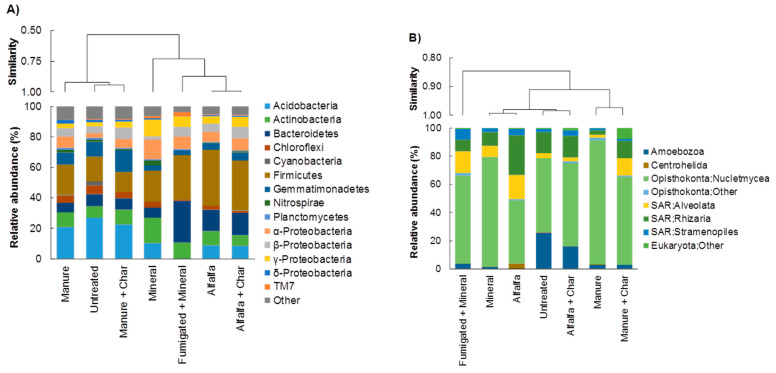
Relative abundance of (**A**) bacterial and (**B**) eukaryotic phyla in soils subjected to different treatments. Dendrogram based on Pearson correlation coefficient represents microbial similarity among treatments.

The relative abundance of bacterial and eukaryotic community composition under each soil treatment (ST) is reported in Figure 2. Considering bacterial composition, hierarchical clustering separates the STs into two main groups (Figure 2A): the first included Untreated, Manure, and Manure + Char treatments. Here, the relative abundances of Acidobacteria, Chloroflexi, Gemmatimonadetes, and δ-Proteobacteria were higher than in other STs, whereas γ-Proteobacteria was the less abundant phylum (Figure 2A). In the second group, Alfalfa and Alfalfa + Char treatments showed a similar bacterial composition, with Firmicutes showing the highest relative abundance. Compared with other treatments, Fumigated + Mineral showed the highest relative abundance of Bacteroidetes, whereas α– and γ–Proteobacteria were the most abundant phylum in Mineral-treated soil (Figure 2A). Considering eukaryotic community composition, hierarchical clustering separates the STs into two main groups (Figure 2B). Generally, members belonging to Nucletmycea were the most abundant, followed by Rhizaria, Alveolata, and Amoebozoa (Figure 2B).

**Figure 3:**
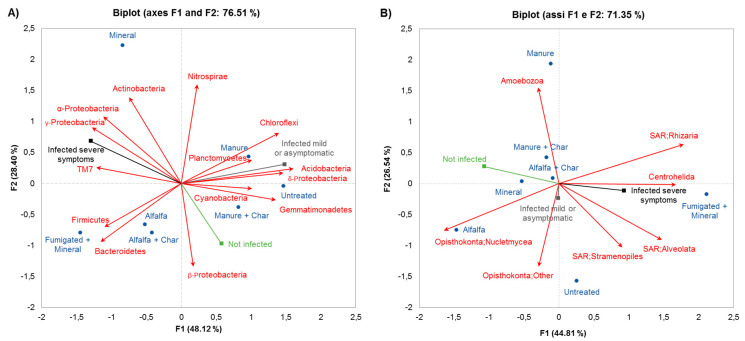
Principal component analysis (PCA) of (**A**) bacterial and (**B**) eukaryotic phyla (red vectors) recorded in different treatments in relation to response of TSWV infection. TSWV-inoculated plants were considered as: (i) infected with severe symptoms (collapsing or dead) (black vector, indicated as severe), (ii) infected with mild symptoms (stunting and or mosaic) or asymptomatic (grey vector, indicated as mild), and (iii) not infected (green vector). These three classes were plotted as supplementary variables following Legendre and Legendre [35].

STs and microbial community composition at the phylum level resulted from principal component analysis (PCA) were simultaneously represented in the correlation biplot (Figure 3A,B). Disease incidence expressed as percentage of infected plants with severe symptoms, infected plants with mild symptoms or asymptomatic, and not infected plants were plotted in the form of vectors as supplementary variables. The PCA provided a satisfactory ordination of the microbial community composition across STs, with the eigenvalues of the first two components accounting for 76.51% (48.12% and 28.40% for F1 and F2, respectively) and 71.35% (44.81% and 26.54% for F1 and F2, respectively) of the total variance for bacteria and eukarya, respectively. Actinobacteria, α-Proteobacteria, γ–Proteobacteria, and TM7 members showed a positive correlation with mortality of infected plants, whereas a negative correlation was observed with δ–Proteobacteria (Figure 3A). Conversely, infected plants with mild symptoms or asymptomatic were positively correlated with Acidobacteria, Chloroflexi, Cyanobacteria, and Planctomycetes, and negatively with Firmicutes and Bacteroidetes (Figure 3A). On the other hand, not infected plants were positively correlated with β- and δ-Proteobacteria, and negatively with Actinobacteria, α– and γ–Proteobacteria, and TM7 (Figure 3A). Among eukarya, not infected plants were positively correlated with Amoebozoa and negatively with Centrohelida and members of the SAR supergroup (including Stramenopiles, Alveolates, and Rhizaria), whereas an opposite correlation pattern was recorded for infected plants with severe symptoms (Figure 3B).

**Figure 4:**
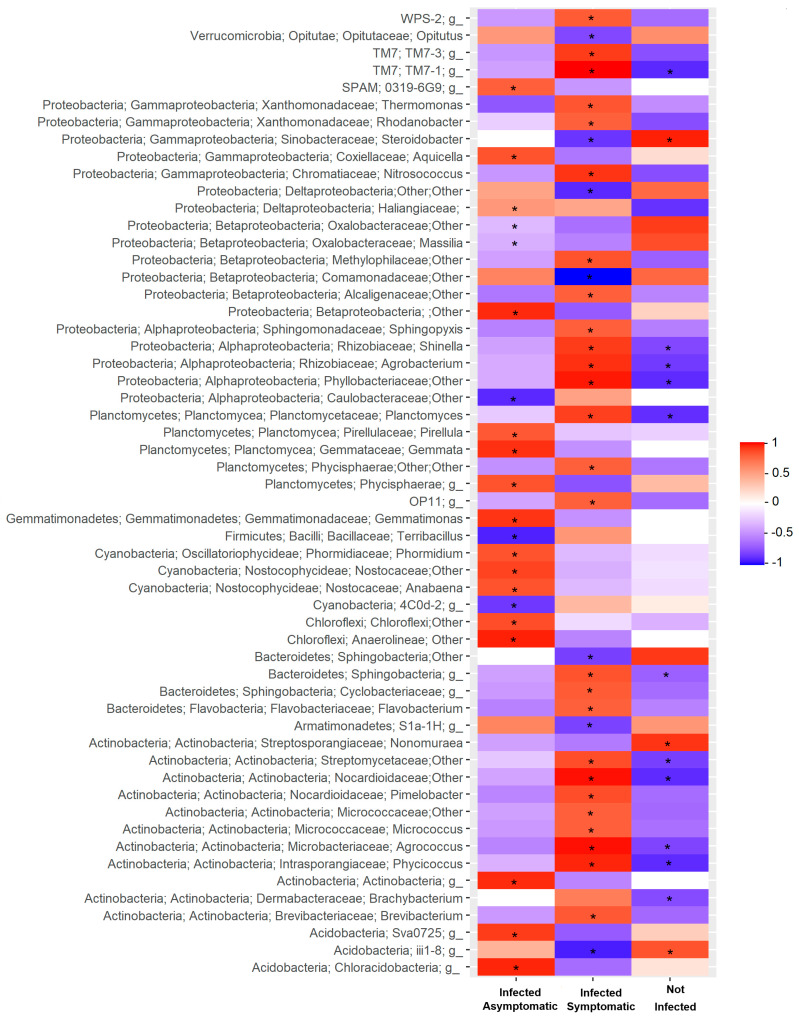
Heat-plot of correlation (Pearson’s r) between bacterial relative abundance collapsed at the genus level with the percentage of tomato plants resulted not infected (ELISA-negative), infected with mild symptoms (stunting and/or mosaic) or asymptomatic, and infected with severe symptoms (collapsing or dead). Only correlations with taxa abundance > 0.1% are reported. Asterisks indicate statistically significant correlation values, negative or positive, at the 0.05 alpha-level of confidence.

**Figure 5:**
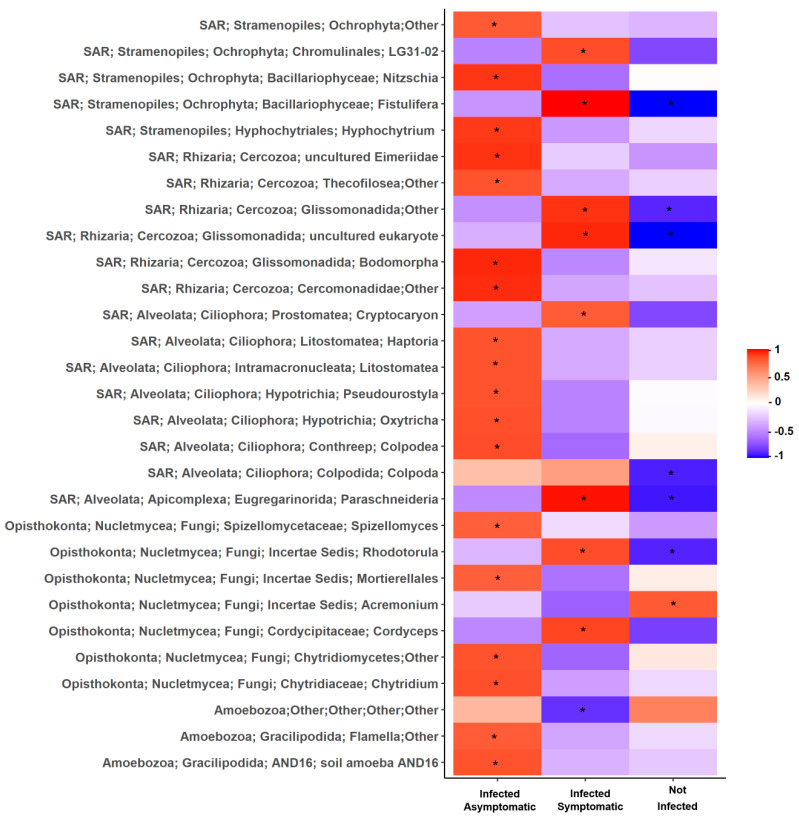
Heat-plot of correlation (Pearson’s r) between bacterial relative abundance collapsed at the genus level with the percentage of tomato plants resulted not infected (ELISA-negative), infected with mild symptoms (stunting and/or mosaic) or asymptomatic, and infected with severe symptoms (collapsing or dead). Only correlations with taxa abundance > 0.1% are reported. Asterisks indicate statistically significant correlation values, negative or positive, at the 0.05 alpha-level of confidence.

Pearson correlation between microbial composition collapsed at the genus level (abundance > 0.1%) and plant response to virus inoculation are reported in Figure 4 and Figure 5 for bacterial and eukaryotic taxa, respectively. The analysis showed that, among the bacteria genera, only 28% (62 out of 220 correlations) were significantly correlated with plant response to virus inoculation, particularly those belonging to Actinobacteria and Proteobacteria phyla. In particular, members of *Massilia, Nonomuraea*, and *Steroidobacter* were positively correlated with the absence of virus infection (ELISA-negative). Conversely, the abundance of several genera, including *Brevibacterium, Phycicoccus, Flavobacterium, Planctomyces, Agrobacterium*, and *Rhodanobacter*, showed a positive correlation with the presence of infected plants with mild symptoms or completely asymptomatic, or with infected plants with severe symptoms (Figure 4).

Considering eukarya, 25% of correlations (29 out of 118) between genera abundance and plant response to virus inoculation were found to be statistically significant (Figure 5). Among these, only the fungal genus *Acremoniun* was positively correlated to the absence of infection. Differently, the fungi *Chytridium*, *Cordyceps, Mortierellales, Rhodotorula*, and *Spizellomyces*, as well as several members of the SAR supergroup like *Paraschneideria, Litomostomatea, Bodomorpha*, and *Nitzschia*, were positively related with the percentage of infected plants showing mild symptoms or asymptomatic, or with infected plants with severe symptoms (Figure 5).

## 3. Discussion

The use of organic amendments to reduce disease incidence of a wide range of soil-borne pathogens is well established, with suppressive effects demonstrated for bacteria (e.g., *Ralstonia solanacearum*, *Streptomyces scabies*), oomycetes (e.g., *Phytophthora* spp., *Pythium* spp.), and fungi (e.g., *Fusarium* spp., *Rhizoctonia solani*, *Rosellinia necatrix*, *Sclerotinia* spp., and *Verticillium dahliae*) [19,36,37,38]. In contrast, only a few studies have reported the effects of OA applications on plant diseases caused by viruses. Aliyu et al. [39] found that soil incorporation of rice-husk significantly reduced the disease incidence of Cowpea mottle virus in cowpea compared to the not amended control. Moreover, the application of 10 tons ha^−1^ of poultry manure significantly reduced the disease incidence and severity of Pepper veinal mottle virus in pepper [40]. However, none of these studies described the impact of organic amendment on soil microbiota nor discussed the potential mechanisms responsible for disease reduction.

In our study, the repeated applications of organic amendments were found to profoundly change bacterial and fungal microbiome that was associated with a substantial reduction of TSWV disease incidence in tomato plants. Specifically, organically managed soils, applied singly or in combination with biochar, caused a lower percentage and a reduced symptom severity of TSWV compared to soils treated with mineral fertilizers and fumigants. In order to adapt OA applications to current agricultural practices, previous studies evaluated the effects of a single initial OA application, demonstrating that it first caused a burst of microbial activity, and then fluctuations, determining instability in soil functionality [41,42]. The biological effect of such single treatment is an initial burst of microbial activity followed by fluctuations that cause instability in soil functionality [41,42]. However, frequent applications of OAs promoted enzymatic activities [43], increased microbial biomass activity [44], and determined soil fungistasis towards a range of soil-borne plant pathogens (i.e., *Aspergillus niger*, *Botrytis cinereal*, and *Pyrenochaeta lycopersici*) [45]. Cesarano et al. [34], using the same soils of the present experiment, reported that the use of a synthetic fertilizer had a higher crop yield, but negatively affected soil quality, causing acidification, salinization, a reduction of soil organic carbon content, and a disruption of soil aggregates. In fact, the repeated applications of chemically different amendments provided a more continuous flux of easily decomposable compounds that sustained soil functioning and enhanced competition for resources, thus resulting in an increased soil biological activity and fungistasis [45]. Here, we found that frequent organic amendment applications have the potential to reduce the disease caused by a plant virus.

Several studies have demonstrated that the direct release of fungitoxic compounds from decaying organic matter [18] increased the competition for nutrients in rhizosphere [20], promoted a direct parasitism [24], and enhanced beneficial microbial antagonism by the release of secondary metabolites [25]. However, none of these effects could explain the suppression or reduction of virus diseases. Besides, it is well known that OAs, like compost and biochar, may suppress airborne fungal pathogens by inducing a systemic resistance (ISR) response [46], as reported for *B. cinerea*, *Leveillula taurica*, and *Podosphaera aphanis* [47,48]. In our experiments, the spatial separation between OA applications and TSWV infection sites allowed us to hypothesize the activation of ISR mechanisms. It is well known that plant beneficial bacteria and fungi can act indirectly by activating latent defense mechanisms that protect epigean plant organs from airborne pathogen attacks [26]. Several studies have demonstrated that beneficial bacteria can reduce plant diseases caused by viruses. Kandan et al. [49] showed that a *P. fluorescens* strain was able to control TSWV on tomato plants. More recently, a soil inoculated with *Paenibacillus lentimorbus* extenuated the virulence of Cucumber mosaic virus in *N. tabacum* [50]. Other studies have demonstrated that different *Bacillus amyloliquefaciens* strains were able to modulate ISR and thus reduce the incidence of tomato diseases caused by Yellow leaf curl virus [51], TSWV, and Potato virus Y [52]. In these works, *Bacillus*-mediated ISR was confirmed by extensive transcriptomics, and histologic and physiological analyses. However, in all these studies, the selected beneficial bacteria were applied to the soil and showed direct induction of systemic resistance to achieve virus biocontrol.

Here, we focused on the complex changes occurring in soil microbiome following OA applications, which may suppress TSWV disease in tomato plants. Cesarano et al. [34] showed that soils managed with organic amendments and mineral fertilizers exhibited a quite different microbiome structure, made by thousands of coexisting bacterial and fungal species. It is noteworthy that diversity and richness of bacteria and eukarya were significantly lower in synthetic- rather than in organic-amended soils [34]. In our study, correlation analyses demonstrated that several taxa were significantly correlated with disease suppression, while others were associated with high virus disease incidence and severity. Notably, in soil treated with organic amendments, a strong correlation between disease suppression and abundance of *Sphingobacteria* and *Massilia* genera was found. Furthermore, Yin et al. [53] reported that the presence of *Massilia* species in rhizosphere protected wheat plants from the attack of *Rhizoctonia solani*.

We also found an association between the abundance of *Acremonium* species and the low TSWV disease frequency and severity. *Acremonium* spp. are commonly reported as endophytic fungi, especially in grasses, with some ability of biological control [54,55]. Conversely, a negative correlation between TSWV disease and abundance of Actinobacteria, *Pseudomonas* spp., and *Agrobacterium* spp., including well-known biocontrol agents [13], was observed. However, our results, based mainly on correlation analyses, do not provide a causal relationship between microbial taxa relative abundance and disease suppression. In this regard, the case of Alfalfa treatment is notable because it showed similar bacterial composition with soil treated with Fumigation + Mineral fertilizer and, in case of eukarya, with mineral application alone. In addition, Alfalfa treatment was one of the most suppressive towards disease caused by TSWV. This contradiction could be at least partially explained based on the difference in soil chemistry, with Fumigated + Mineral and Mineral treatments that caused soil acidification, increase of salinity, and a dramatic reduction of biological acidity assessed by the BIOLOG Eco-Plates™ method [34]. Overall, these results support the hypothesis that suppressiveness mediated by organic amendments is related to an overall shift in soil quality in terms of microbiome composition, functionality, and chemistry. In other words, we speculate that the functional diversity of bacteria and eukarya is more relevant than the absence or presence of specific taxa. This assumption is in agreement with the emerging understanding about soil suppressiveness [23].

Biochar have been used since ancient times as soil amendments and, more recently, in plant disease suppression [19]. However, several factors, including the pyrolysis temperature, the plant-pathogen systems, and the type and quality of the initial organic feedstock, may affect biochar chemistry, structure, and biological activity [56]. Our results indicated that biochar combined with alfalfa or manure were among the most effective soil treatments to control the disease caused by TSWV on tomato plants, thus suggesting the possibility to develop novel biocontrol products based on biochar appropriately combined with selected non-pyrogenic OAs. Recently, Bonanomi et al. [57] reported that biochar mixed with N-rich and lignin-poor materials (i.e., alfalfa hay and manure) acted synergistically to promote plant growth. The mechanisms that may explain the synergistic effects include the improved water availability and soil structure [58], as well as the enhanced availability of mineral nutrients [59]. Kammann et al. [60] demonstrated that co-composted biochars became enriched with nutrients (i.e., ammonium, nitrate, and phosphate), as well as with dissolved organic carbon. As for diseases’ suppression, here, we speculated that biochar mixed with non-pyrogenic amendments was complementary in supporting microbial life. The biochar may provide soil porous structures that physically protect microbial colonies from grazers or predators, like mites, protozoans, collembola, and nematodes [61,62]. Postma et al. [63] demonstrated that *Bacillus pumilus, Pseudomonas chlororaphis*, and *Streptomyces pseudovenezuelae* colonized biochar pores and provided an effective control to tomato diseases caused by *Pythium aphanidermatum* and *F. oxysporum* f. sp. *lycopersici*. On the other hand, the non-pyrogenic fraction (i.e., alfalfa hay or manure) provided food substrates to sustain the activity of biocontrol agents [20], that are important for soil and rhizosphere colonization. In this regard, the variability in the suppressive capacity of OAs and the lack of its predictability largely limit the degree of commercial application of non-pyrogenic organic amendments [19]. A recent study demonstrated that biochar pre-conditioning with soil nutrient solution enhanced the suppression of damping-off caused by *Pythium aphanidermatum* through the promotion of beneficial microbiota [64]. Overall, available evidence suggests the possibility of developing novel biocontrol products that, by combining biochar with appropriate non-pyrogenic organic amendment, may have a more consistent suppression against a range of plant pathogens.

The characterization and selection of the appropriate chemical composition of non-pyrogenic organic materials is necessary to promote the effect of biocontrol agents or beneficial microbiomes, as well as to improve natural soil suppressiveness against soil-borne pathogens [19]. Actually, the combination of biochar with non-pyrolyzed organic materials (i.e., compost, manure, plant residues) represents the active ingredient of several commercial formulations, generally referred to as “*terra preta*”-like planting substrates [65].

## 4. Materials and Methods

### 4.1. Soil Treatments and Microbial Analysis

Soil used in this study was collected from a farm located in the Salerno area, Southern Italy, as described previously [34]. Plastic trays (32 L) were filled with soil, placed in a greenhouse equipped with automatic control of temperature, and subjected to different soil treatments (STs). These consisted of: (1) the application of synthetic fertilizers (Mineral), (2) soil fumigation with Metham-Na plus application of synthetic fertilizers (Fumigated + Mineral), (3) application of compost manure (Manure), (4) application of compost manure plus wood biochar (Manure + Char), (5) application of glucose and alfalfa straw (Alfalfa), and (6) application of glucose and alfalfa straw plus wood biochar (Alfalfa + Char). Untreated soil served as the control (Untreated). Details about doses and application frequency were described by Cesarano et al. [34]. Each treatment was replicated three times for a total of 21 plastic trials. During the mesocosm experiment (1 year), rocket (*Eruca sativa*) was cultivated six times.

At the end of the first conditioning year, soil microbiomes were analyzed by Illumina high-throughput sequencing techniques and results were described as reported in Cesarano et al. [34]. Here, the data were used to explore the effect of bacterial and eukaryotic community composition on TSWV incidence and symptom expression. The 16S and 18S rRNA gene sequences from our previous work are available at the Sequence Read Archive (SRA) of the National Center for Biotechnology Information (NCBI), under accession number SRP092020.

### 4.2. TSWV-Tomato Experiment

At the end of the conditioning experiment that lasted one year, the soils subjected to the different STs were air-dried for three weeks and, thereafter, used to fill sterilized pots (16 cm diameter, 20 cm height). Overall, seven soil types derived from different STs were used: (1) Mineral, (2) Fumigated + Mineral, (3) Manure, (4) Manure + Char, (5) Alfalfa, (6) Alfalfa + Char, and (7) Untreated. The experiment was conducted in a thermo-conditioned greenhouse with temperature kept at 20 ± 2 °C (night) and 24 ± 2 °C (day). Pots were watered to 80% of field capacity. One tomato (*Solanum lycopersicum*, L.) seedling (cv. Roma) was transplanted in each pot and allowed to interact with the soil microbiota for ten days. Subsequently, tomato plants were inoculated with TSWV, isolate PI-S. The isolate PI-S was obtained from a tomato plant collected in Salerno province (Southern Italy), and propagated from local lesions induced on the primary leaves of *Nicotiana tabacum* (cv. Samsun). The isolate was maintained in plants of *N. glutinosa*, where the inoculum was purified by several serial inoculations [66]. Briefly, leaves (1 g) of *N. glutinosa* systemically infected by TSWV isolate PI-S were ground in 10 mL of potassium phosphate buffer (10 mM), pH 7.0–7.2, using a pestle and mortar. Tomato plants were dusted with abrasive carborundum (Fisher Scientific) and cotton swabs dipped in the inoculum were rubbed on cotyledons and early leaves of plants. After inoculation, the plants were sprayed with tap water to remove carborundum from the leaf surface. In control plants, the same procedure was performed by using only phosphate buffer as inoculum. During the experiment, pots were watered every 2–3 days to maintain soil moisture content between 60% and 80% of field capacity. The experimental setup included a total of 7 STs and consisted in 18 replicates for TSWV-infected plants and 6 replicates for controls, resulting in a total of 168 pots. Pots were arranged in the greenhouse in a completely randomized block design.

### 4.3. Assessment of TSWV Infection

Symptoms like stunting, mosaic, collapsing, and plant death were recorded 21 days after virus inoculation. In addition, three weeks after inoculation, all tomato plants were assayed by enzyme-linked immunosorbent assay (ELISA) [67], regardless of the presence of disease symptoms. ELISA was carried out by grounding tissues in phosphate buffered saline with Tween 20 (1/10, *w/v*), and the extracts were tested using a commercial TSWV ELISA diagnostic kit (Loewe Biochemica GmbH, Germany). Disease severity was recorded as percentage infection in inoculated plants according to the detection of TSWV by ELISA at OD 405 nm. Plants were classified, according to visual assessment of symptoms and ELISA results, in four classes: (i) not infected (ELISA-negative) plants, (ii) infected (ELISA-positive) and asymptomatic plants, (iii) infected plants (ELISA-positive) with mild symptoms (stunting and/or mosaic), and (iv) infected plants (ELISA positive) with severe symptoms (collapsing or death).

### 4.4. Statistical Analysis

The effect of different STs on TSWV incidence was expressed as the percentage of infected plants. Data were subjected to one-way analysis of variance (ANOVA) and significant differences between treatments were determined by using Duncan’s multiple range test at the 0.05 alpha-level of confidence. Data of soil microbiomes and the TSWV-tomato experiment were submitted to multivariate principal component analysis (PCA) in order to assess the multiple relationships among bacterial and eukaryotic community composition with disease incidence. For PCA, three classes were considered: (i) not infected (ELISA-negative) plants, (ii) infected (ELISA-positive) plants, both asymptomatic and with mild symptoms, and (iii) infected (ELISA-positive) plants, with severe symptoms. These three classes were plotted as loading vectors in the bi-dimensional PCA space even if it was not used to compute the eigenvalues of the same ordination space, following the approach reported by Legendre and Legendre [35]. Finally, to address the relationship between soil microbial structure and response to virus inoculation, Pearson linear correlation was calculated between microbial relative abundance and the percentage not infected, infected—both asymptomatic and with mild symptoms, and infected plants with severe symptoms. Correlation analysis was carried out for bacterial and eukaryotic community collapsed at the genus level with abundance > 0.1% and tested for statistical significance at the 0.05 alpha-level of confidence. All statistical analyses were performed by using STATISTICA 14 software (StatSoft Inc., Tulsa, OK, USA).

## 5. Conclusions

In conventional farming, the extensive use of fumigants and mineral fertilizers negatively affects soil microbiome diversity [9,68] and functionality [69]. A recent study pointed out that the repeated soil sterilizations with the chemical fumigant Metham-Na disrupted the soil food web, impairing disease suppression capability towards *Rhizoctonia solani* [70]. The present study confirms that mineral fertilizers alone, or in combination with Metham-Na, may shape a completely different microbiome than that observed under repeated applications of OAs. As a consequence of this microbiome shift, a dramatic increase of TSWV disease incidence and severity was observed, although the causal relationship between these observations is still unclear. It is noteworthy that TSWV is a significant problem in intensive conventional farming systems [29], that are subjected to periodical sterilization treatments (e.g., fumigation with different chemicals) combined with large applications of mineral fertilizers. Our work suggests that conventional management practices may have harmful effects on soil microbiome structure and functions that, in turn, may result in a reduction in plant resistance to TSWV. Further studies, however, are needed to elucidate the complex link between microbiome functioning, soil management practices, plant resistance, and disease suppression towards viruses.

## Supplementary Materials

The following are available online at https://www.mdpi.com/2076-0817/9/5/379/s1: Table S1: Summary of the four one-way ANOVA tests analyzing the main effects of soil treatments on TSWV incidence, based on four classes: (i) not infected (ELISA-negative), (ii) infected (ELISA-positive) and asymptomatic plants, (iii) infected plants (ELISA-positive), with mild symptoms (stunting and/or mosaic), and (iv) infected plants (ELISA-positive) with severe symptoms (collapsing or death). Table S2: One-way ANOVA, followed by post hoc Duncan’s test for the four plant categories: not infected (ELISA-negative), infected (ELISA-positive) and asymptomatic plants, infected plants (ELISA-positive) with mild symptoms (stunting and/or mosaic), and infected plants (ELISA-positive) with severe symptoms (collapsing or death). Table S3: Mean absorbance values at OD 405 nm obtained by the ELISA test. Table S4: One-way ANOVA, followed by post hoc Duncan’s test for the data obtained by the ELISA test.
