# Supplementary material for: Organic Amendments Modulate Soil Microbiota and Reduce Virus Disease Incidence in the TSWV-Tomato Pathosystem
Source: Pathogens. 2020 May 14;9(5):379. doi: 10.3390/pathogens9050379 (PMC7281679; doi:10.3390/pathogens9050379)
Supplement: Supplementary file 1 [file pathogens-09-00379-s001.pdf]

Supplementary Material

Effect	Sum of Squares	Degree of freedom	Mean Squares	F-Distribution	P-Value
**Not infected**					
**Soil treatment**	216.74	6	36.12	12.74	**< 0.001**
**Infected and asymptomatic**					
**Soil treatment**	6.09	6	1.01	4.95	**< 0.001**
**Infected with mild symptoms**					
**Soil treatment**	8.52	6	1.42	7.42	**< 0.001**
**Infected with severe symptoms**					
**Soil treatment**	12.38	6	2.06	24.69	**< 0.001**

Supplementary Table 1. Summary of the four One-way ANOVA testing for main effect of soil treatments on TSWV incidence based on four classes: not infected (ELISA negative), infected (ELISA positive) and asymptomatic plants; infected plants (ELISA positive) with mild symptoms (stunting and/or mosaic), infected plants (ELISA positive) with severe symptoms (collapsing or death). Significance of *P*-values was determined with 0.05 alpha-level of confidence.

Variable: Infected plants (ELISA positive) with severe symptoms

	Untreated	Mineral	Fumigated + Mineral	Manure	Alfalfa	Manure + Char	Alfalfa + Char
**Untreated**		0.0001	0.0001	0.2055	1.0000	0.2176	0.1841
**Mineral**	0.0001		1.0000	0.0000	0.0000	0.0000	0.0001
**Fumigated + Mineral**	0.0001	1.0000		0.0000	0.0001	0.0000	0.0002
**Manure**	0.2055	0.0000	0.0000		1.1841	1.0000	0.0209
**Alfalfa**	1.0000	0.0000	0.0001	0.1841		0.2055	0.2055
**Manure + Char**	0.2176	0.0000	0.0000	1.0000	0.2055		0.0229
**Alfalfa + Char**	0.1841	0.0001	0.0002	0.0209	0.2055	0.0229	

Variable: Infected plants (ELISA positive) with mild symptoms

	Untreated	Mineral	Fumigated + Mineral	Manure	Alfalfa	Manure + Char	Alfalfa + Char
**Untreated**		0.0002	0.0000	0.0000	0.0000	0.0001	0.0001
**Mineral**	0.0002		0.0037	0.0000	0.0033	0.0937	0.0800
**Fumigated + Mineral**	0.0000	0.0037		0.0022	1.0000	0.0937	0.1023
**Manure**	0.0000	0.0000	0.0022		0.0028	0.0002	0.0001
**Alfalfa**	0.0000	0.0033	1.0000	0.0028		0.0800	0.0937
**Manure + Char**	0.0001	0.0937	0.0937	0.0002	0.0800		1.0000
**Alfalfa + Char**	0.0001	0.0800	0.1023	0.0001	0.0937	1.0000	

Variable: Infected (ELISA positive) and asymptomatic plants

	Untreated	Mineral	Fumigated + Mineral	Manure	Alfalfa	Manure + Char	Alfalfa + Char
**Untreated**		0.0092	0.0082	0.0001	0.0055	0.1223	0.0070
**Mineral**	0.0092		1.0000	0.0000	1.0000	0.0005	1.0000
**Fumigated + Mineral**	0.0082	1.0000		0.0000	1.0000	0.0004	1.0000
**Manure**	0.0001	0.0000	0.0000		0.0001	0.0002	0.0000
**Alfalfa**	0.0055	1.0000	1.0000	0.0001		0.0004	1.0000
**Manure + Char**	0.1221	0.0005	0.0004	0.0002	0.0004		0.0004
**Alfalfa + Char**	0.0070	1.0000	1.0000	0.0000	1.0000	0.0004	

Variable: Not infected plants (ELISA negative)

	Untreated	Mineral	Fumigated + Mineral	Manure	Alfalfa	Manure + Char	Alfalfa + Char
**Untreated**		0.0001	0.0009	0.0009	0.0000	0.0002	0.0001
**Mineral**	0.0001		0.0516	0.0001	0.0000	0.0000	0.0000
**Fumigated + Mineral**	0.0009	0.0516		0.0001	0.0000	0.0001	0.0000
**Manure**	0.0009	0.0001	0.0001		0.0013	0.3051	0.0617
**Alfalfa**	0.0000	0.0000	0.0000	0.0013		0.0085	0.0516
**Manure + Char**	0.0002	0.0000	0.0001	0.3051	0.0085		0.3051
**Alfalfa + Char**	0.0001	0.0000	0.0000	0.0617	0.0516	0.3051	

Supplementary Table 2. ANOVA one-way, followed by post hoc Duncan's test for the four plant categories: infected plants (ELISA positive) with severe symptoms (collapsing or death); infected plants (ELISA positive) with mild symptoms (stunting and/or mosaic); infected (ELISA positive) and asymptomatic plants; not infected plants (ELISA negative). Significance of *P*-values, highlighted in red, was determined with 0.05 alpha-level of confidence.

Treatment	OD 405
Alfalfa + Char	0.22±0.21b
Manure + Char	0.29±0.17b
Alfalfa	0.11±0.09a
Manure	0.31±0.18b
Fumigated + Mineral	0.27±0.21b
Untreated	0.38±0.29b
Mineral	0.24±0.18b

Supplementary Table 3. Mean absorbance at OD405 nm obtained by ELISA test. Data were submitted to one-way ANOVA to evaluate the main effects of soil treatments on viral titre. Values are average ± standard deviation of 18 replicates, different letters indicate significant differences (Duncan's test, significance determined with 0.05 alpha-level of confidence).

	**Alfalfa**	**Alfalfa + Char**	**Fumigated + Mineral**	**Manure**	**Manure + Char**	**Mineral**	**Untreated**
**Alfalfa**		**0.000057**	**0.000037**	**0.000022**	**0.000027**	**0.000148**	**0.000064**
**Alfalfa + Char**	**0.000057**		**0.916635**	**0.558756**	**0.636379**	**0.609737**	**0.912797**
**Fumigated + Mineral**	**0.000037**	**0.916635**		**0.609737**	**0.688362**	**0.558756**	**0.841550**
**Manure**	**0.000022**	**0.558756**	**0.609737**		**0.884718**	**0.302341**	**0.505684**
**Manure + Char**	**0.000027**	**0.636379**	**0.688362**	**0.884718**		**0.356126**	**0.580431**
**Mineral**	**0.000148**	**0.609737**	**0.558756**	**0.302341**	**0.356126**		**0.662486**
**Untreated**	**0.000064**	**0.912797**	**0.841550**	**0.505684**	**0.580431**	**0.662486**	

Supplementary Table 4. ANOVA one-way, followed by post hoc Duncan's test for the data obtained by ELISA test. Significance of *P*-values, highlighted in red, was determined with 0.05 alpha-level of confidence.
